# Tailored Target Ablation Index Guided Pulmonary Vein Isolation in Treating Paroxysmal Atrial Fibrillation: A Single Center Randomized Study in Asian Population (AI-Asian-I)

**DOI:** 10.3389/fcvm.2022.937913

**Published:** 2022-07-07

**Authors:** Qingsong Xiong, Jia Liao, Weijie Chen, Peilin Xiao, Huaan Du, Qushuai He, Yuehui Yin, Zhiyu Ling, Shaojie Chen

**Affiliations:** Department of Cardiology, Second Affiliated Hospital of Chongqing Medical University, Chongqing, China

**Keywords:** atrial fibrillation, ablation index, pulmonary vein isolation, randomized, recurrence

## Abstract

**Objective:**

To evaluate the efficacy and safety of lower ablation indexes (AI) guided pulmonary vein isolation (PVI) in treating paroxysmal atrial fibrillation (AF).

**Methods:**

Ninety patients with paroxysmal AF scheduled for radiofrequency ablation were randomly divided into three groups. The AI targets for PVI were as follows: In group A/B/C, 550/500/450 for roof and anterior wall, and 400/350/300 for posterior/inferior wall. The first-pass PVI rate, ablation time, complications and recurrence of atrial tachyarrhythmia (ATa) were compared.

**Results:**

The mean age was 62.5 years (male: 63.0%), mean body mass index (BMI): 24.35 ± 3.66 kg/m^2^. The baseline characteristics were comparable. There was no significant difference in the first-pass PVI rate among the three groups (left-sided-PV: 66.7% vs. 80% vs. 73.3%, *P* = 0.51; right-sided-PV: 70% vs. 83.3% vs. 73.3%, *P* = 0.64), also with similar gap rate during the procedural waiting time. At 1-year follow-up there was no significant difference in the recurrence rate of ATa among the three groups (10% vs. 13.3% vs. 13.3%, *P* = 1.00). The ablation time in the Group C was significantly less than that in the other two groups (47.8 min. vs. 47.0 min. vs. 36.6 min, *P* < 0.001). Higher AI seemed to link a non-significant trend toward higher rate of pericardial effusion (group A + B vs. group C:6.7% vs. 0%, *P* = 0.30), although the rate of overall complications was not different among the three groups.

**Conclusion:**

This randomized study shows that, a relatively lower target AI guided ablation may be similarly effective to achieve PVI with significantly reduced ablation time and obtain similar clinical outcome in treating paroxysmal AF in Asian population.

**Clinical Trial Registration:**

[www.ClinicalTrials.gov], identifier [NCT:04549714].

## Introduction

Catheter ablation is an effective treatment option for patients with symptomatic atrial fibrillation (AF) (1–5). Electrical reconnection between the pulmonary veins and left atrium has been recognized as an important factor responsible for AF recurrence after ablation (6, 7). Radiofrequency ablation is conventionally performed with or without contact-force technology, usually not guided by standardized quantitative criteria. Lower ablation energy delivery may result in non-transmural damage, but over-shooting increases ablation time and may increase risk of complications (8, 9).

The ablation index (AI) which integrates catheter contact force, ablation power, and ablation duration is an algorithm to guide radiofrequency ablation, and the AI can be used to quantify the ablation energy and predict lesion formation (10–12). Previous studies have shown the efficacy of AI guided ablation for AF, and the target AI values adopted are generally 550 for the anterior wall and the roof and 400 for the inferior/posterior wall (13, 14).

However, (1) the target AI 550/400 was mainly obtained from European-American population; (2) The lesion depth caused by ablation guided by target AI of 550/400 can be deeper than the atrial thickness *per se* and may therefore potentially increase the risk of complications, particularly among Asian population, an ethnic group normally with smaller atrial size (15, 16). The aim of this randomized study was to investigate the feasibility, safety, efficacy and clinical outcome of catheter ablation guided by predefined lower target AI for pulmonary veins isolation (PVI) in treating paroxysmal AF in Asian population.

## Materials and Methods

### Study Design

**FIGURE 1:**
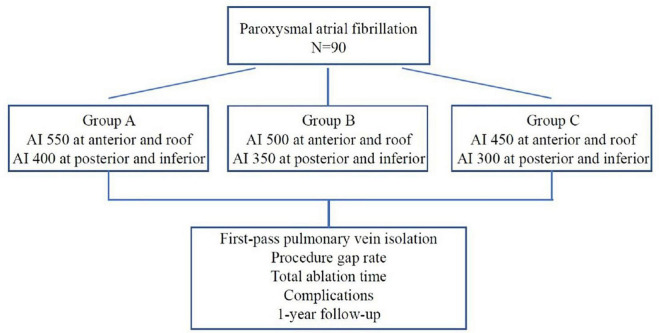
The study flowchart.

The study was designed as a prospective randomized, single blind, non-placebo controlled clinical study with the primary objective of assessing the optimal ablation index to achieve effective pulmonary vein isolation. A total of 90 patients with paroxysmal AF were randomly assigned to group A, B, and C by using a randomization envelope. Group A was designed with target AI value of 550 at the roof and anterior wall and 400 at the posterior and inferior wall; Group B was designed with target AI value of 500 at the roof and anterior wall and 350 at the posterior and inferior wall; Group C was designed with target AI value of 450 at the roof and anterior wall, and 300 at the posterior and inferior wall (Figure 1). The study was approved by ethics committee of Second Affiliated Hospital of Chongqing Medical University and complied with the declaration of Helsinki. Written informed consent was obtained from all patients.

### Study Population and Exclusion

Study population: (1) Patients with symptomatic paroxysmal AF; (2) Age 18–80 years; (3) Without contraindication to anticoagulant therapy; (4) Transoesophageal ultrasonography excluded atrial/left atrial appendage thrombi. Exclusion criteria: (1) Patients who had undergone prior catheter ablation for AF; (2) Left ventricular ejection fraction (LVEF) < 35%; (3) Pregnant, prepared pregnant or lactating women; (4) Left atrial appendage thrombus detected by transesophageal or intracardiac ultrasonography; (5) Severe abnormalities of hematologic or hepatic and renal function; (6) Combined with other heart disease (congenital heart disease, valvular heart disease, dilated cardiomyopathy, hypertrophic cardiomyopathy); (7) Acute myocardial infarction or patients who had undergone PCI or CABG within 1 year; (8) Pulmonary vein stenosis, occlusion, or thrombus.

### Pre-procedure

Pre-operative Transesophageal ultrasonography was completed to exclude intracardiac thrombi. Cardiac Computed tomography (CT) or magnetic resonance imaging (MRI) was performed to assess the anatomy. All patients received anticoagulant therapy according to the recommendation (17). To reduce the risk of bleeding during ablation, oral anticoagulant drugs were suspended on the day of ablation. All patients discontinued antiarrhythmic drugs 5 half-life period.

### Ablation

**FIGURE 2:**
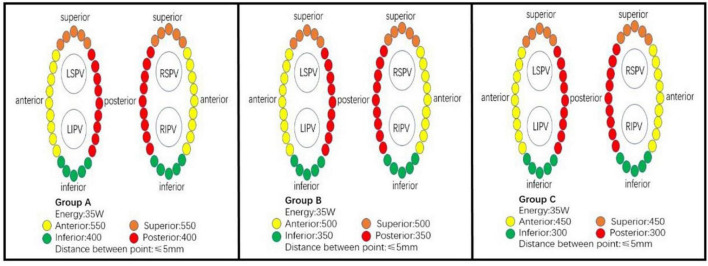
Pulmonary vein segments and ablation protocol in Group A/B/C.

Procedures were performed by two operators; both of them have had more than 5 years of ablation experience and more than 50 procedures per year. All ablation were under local anesthesia, and fentanyl was used to reduce pain during ablation. Heparin was administered at 100–120 U/kg and activated prothrombin time (ACT) was maintained at 300–400 S. After the puncture of the bilateral femoral veins, a 7F vascular sheath was placed through the left femoral vein and a diagnostic electrode catheter (SinusFlex™, APT Medical, China) was placed in the coronary sinus. Two 8.5F Swartz transseptal sheaths (St. Jude) were advanced through the right femoral vein, and transseptal puncture was performed under the guidance of fluoroscopy. A spiral diagnostic mapping catheter (Lasso, Biosense-Webster Inc., Diamond Bar, CA) was positioned in left atrium *via* the transseptal sheath to document the pulmonary vein ostia potential. A 3.5 mm irrigated tip ablation catheter (Thermocool SmartTouch; Biosense-Webster Inc., Diamond Bar, CA) was advanced in the left atrium *via* the other transseptal sheath. Anatomical model of left atrial, pulmonary vein, and mitral annulus was constructed under the guidance of the 3D mapping system (CARTO 3 V6; Biosense-Webster, Inc., Diamond Bar, CA). Wide-area circumferential pulmonary vein antrum isolation (PVI) was performed under the guidance of carto mapping system and automated lesion tagging (VisiTag™, Biosense Webster Inc., DiamondBar, CA, United States). The following Visitag settings were used: 3 mm stability for 3 s. Inter-lesion distance (ILD) targets ≤ 5 mm. Ablation power was set at 35 W, target contact force between 5 and 15 g, irrigation rate of 25 ml/min. The target AI for ablation was determined according to randomization (Figure 2). The ablation endpoint was reached if disappearance (or dissociation) of all pulmonary vein potential after completion of the ablation circles. Otherwise, the gaps were searched and ablated until successful PVI. If patient remained in AF after PVI, electrical cardioversion was performed to restore sinus rhythm. 30 min waiting time was required for all patients after PVI, each pulmonary vein was re-examined to confirm procedural success.

### Follow-Up

All patients returned to the hospital for follow-up at 1, 3, 6, and 12 months after discharge. The main contents included: 12-channel electrocardiography(ECG), 24-h Holter ECGs, and clinical symptoms. Three-month after the procedure was defined as a blank period. Oral antiarrhythmic drugs remained discontinued after the procedure, cardioversion or AADs were allowed if early recurrence during the blanking period. In case of any symptoms suggestive of an arrhythmia recurrence, 24 h Holter ECGs were performed or patient received an external event monitor. Arrhythmia recurrence was defined as ECG documented atrial arrhythmias longer than 30 s after 3 months without AADs. Oral preventive PPI inhibitors were used for 1 month after the ablation. The primary outcomes were the rate of first-pass PVI and clinical recurrence, and secondary outcomes referred to procedural data, including ablation time, procedure time, rate/location of gaps and procedural complications.

### Statistics

The sample size calculation was primarily based on the rates of first-pass PVI guided by different AIs reported in previous literatures, i.e., the rate of first-pass PVI ranged from ca. 70% guided by lower target AI to ca. 95% guided by higher target AI. At α level of 0.05, the sample size of 64 patients (32 in each group) could provide 80% statistical power to estimate the differences between two groups.

Normally distributed continuous data were expressed as mean ± standard deviation. Non-normally distributed data were presented as median with interquartile range. Categorical data were presented as frequencies and percentages. The normality of variable distribution was tested using the Shapiro–Wilk test. The *t*-test was used to compare continuous variables with normal distribution; otherwise the Mann–Whitney test was used. For categorical variables, comparisons between groups were performed using the chi square test or Fisher’s exact test. Kaplan – Meier analysis and log rank test were used for event free survival analysis. For all calculations, two tailed tests were used, and the level of significance was set at a *p*-value of 0.05. All calculations were performed using SPSS 25 (IBM Corp., Armonk, NY, United States).

## Results

### Population Baseline Characteristics

The study recruited the first patient on June 18, 2019 and the last patient on June 29, 2020. All 90 patients completed 1 year follow-up and were included in the data analysis. Table 1 reports the demographic information of all included patients. The three groups were balanced in terms of basic characteristics, mean age 62.5 years, 63.3% male, mean left atrial diameter 36.1 mm by TTE, and left ventricular ejection fraction 68.6%.

**TABLE 1:** Baseline characteristics.

Characteristics	Total (*n* = 90)	Group A (*n* = 30)	Group B (*n* = 30)	Group C (*n* = 30)	*P*-value
Age, years ± *SD*	62.5 ± 10.5	61 ± 10	63 ± 13	63 ± 13	0.74
Male gender, *n* (%)	57 (63.3)	18 (60)	19 (63.3)	20 (66.7)	0.87
Diabetes mellitus, *n* (%)	12 (13.3)	4 (13.3)	5 (16.7)	3 (10)	0.93
Hypertension, *n* (%)	44 (48.9)	11 (36.7)	18 (60)	15 (50)	0.19
Stroke/TIA (including lacunar), *n* (%)	16 (17.8)	4 (13.3)	9 (30)	3 (10)	0.95
Heart failure, *n* (%)	10 (11.1)	2 (6.7)	5 (16.7)	3 (10)	0.59
CHD, *n* (%)	16 (17.8)	5 (16.7)	4 (13.3)	7 (23.3)	0.59
BMI, kg/m^2^, mean ± *SD*	24.35 ± 3.66	25.17 ± 2.78	23.88 ± 4.43	24.01 ± 3.66	0.12
Smoke, *n* (%)	33 (36.7)	10 (33.3)	11 (36.7)	12 (40)	0.87
LA diameter (mm), mean ± *SD*	36.1 ± 3.8	35.9 ± 3.5	35.8 ± 4.4	36.6 ± 3.5	0.45
LVEF (%), mean ± *SD*	68.6 ± 7.0	67.9 ± 6.2	69.2 ± 6.7	68.6 ± 8.1	0.77
CHA_2_DS_2_-VASc, mean ± *SD*	2.2 ± 1.5	1.9 ± 1.4	2.1 ± 1.4	2.7 ± 1.5	0.06

*BMI, body mass index; LA, left atrium; CHD, coronary heart disease; LVEF, left ventricular ejection fraction.*

### First Pass Pulmonary Vein Isolation

As shown in Table 2, The first-pass isolation rate of left-sided pulmonary vein was 73.3% in 90 patients, and there was no significant statistical difference among the three groups (67.7% vs. 80% vs. 73.3, *P* = 0.51). The first-pass isolation rate of the right-sided pulmonary vein was 75.6%, with no significant difference between the three groups (70% vs. 83.3% vs. 73.3, *P* = 0.64) (Table 2).

**TABLE 2:** Comparison of first-pass PVI among three groups.

	Group (AI)	Total (circle)	First-pass PVI (*n*, %)	*P-*value
LPV	Group A (550/400)	30	20 (67.7)	0.51
	Group B (500/350)	30	24 (80)	
	Group C (450/300)	30	22 (73.3)	
RPV	Group A (550/400)	30	21 (70)	0.64
	Group B (500/350)	30	25 (83.3)	
	Group C (450/300)	30	22 (73.3)	

*RPV, Right-sided pulmonary vein; LPV, left-sided pulmonary vein; PVI, pulmonary vein isolation.*

**FIGURE 3:**
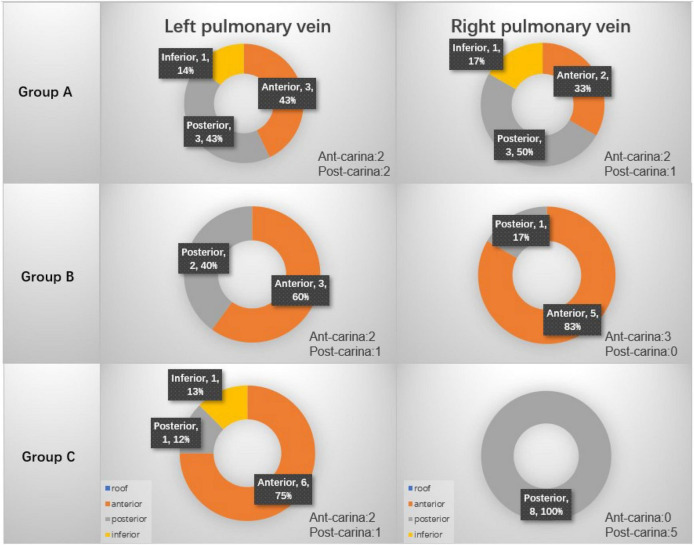
Procedural gap distribution among three groups.

For patients who did not have first-pass isolation, the location of gap was searched. Figure 3 summarizes the procedural gap rate and the distribution of the gaps. In details, for the left pulmonary veins, in group A, 3 gaps were located at the anterior wall, 3 gaps at the posterior wall and 1 gap at the inferior; in Group B, 2 gaps at the posterior wall and 3 gaps at the anterior wall; in group C, there were 6 gaps at anterior wall, 1 gap at inferior wall, and 1 gap at posterior wall. For the right pulmonary veins, in group A, 2 gaps were located at the anterior wall, 3 gaps at the posterior wall, 1 gap at the inferior wall; in Group B, 1 gap at the posterior wall and 5 gaps at the anterior wall; in group C, 8 gaps were at the posterior wall. Table 3 compares the procedural gaps rate (including the 30 min waiting time) among the three groups, and no significant difference was observed.

**TABLE 3:** Comparison of gap rate after first-around ablation circles and during waiting time.

1) Group/segment	Gap/N (per circle) (After first around circles)	*P*
Group A Gap roof/anterior	5/60	0.66
Group B Gap roof/anterior	8/60	
Group C Gap roof/anterior	6/60	
Group A Gap inferior/posterior	8/60	0.12
Group B Gap inferior/posterior	3/60	
Group C Gap inferior/posterior	10/60	
2.) Total gap rate	Gap/N (per circle) (After first around circles)	P
Group A	13/60	0.54
Group B	11/60	
Group C	16/60	
3). Gap rate	Gap/N (per circle) (Waiting time)	P
Group A	0/60	NS
Group B	0/60	
Group C	0/60	

### Recurrence

**FIGURE 4:**
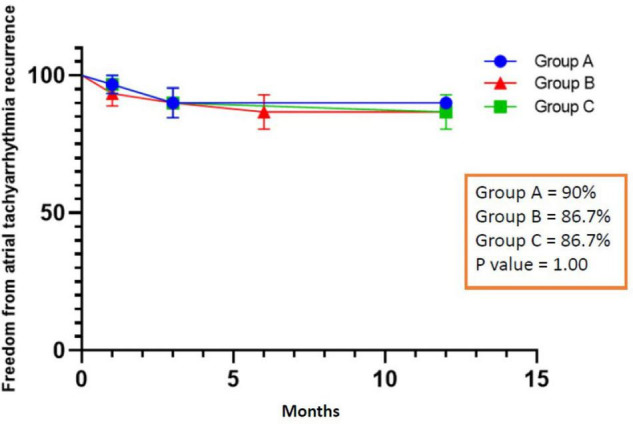
Freedom from atrial tachyarrhythmia recurrence.

All patients completed 1-year follow-up. The results showed that 3 patients had recurrent AF after ablation in group A, 4 patients in group B (1 patient had recurrent left atrial flutter), and 4 patients recurrent AF in group C. The rate of ATa recurrence was similar among the three groups (10% vs. 13.3% vs. 13.3%, *P* = 1.00), and Figure 4 shows the Kaplan Meier Curve for freedom from ATa recurrence. In univariate and multivariate regression analysis, no factor related to the recurrence of ATa was found (Table 4).

**TABLE 4:** Univariate and multivariate Cox regression analysis for ATa recurrence.

Factor	Patients without ATs recurrence	Patients with ATs recurrence	Univarite analysis		Multivariable	
			*P*	HR	*P*	HR
Lower AI (Group C, %)	26 (32.9)	4 (36.4)	0.84	1.06 (0.58–1.97)	0.65	1.17 (0.59–2.32)
Age	62.53	62.95	0.48	1.02 (0.96–1.09)	0.10	1.07 (0.99–1.16)
CHA_2_DS_2_-VASc	2.23	2.27	0.50	0.86 (0.57–1.32)	0.42	0.77 (0.41–1.45)
HAS-BLED	0.92	0.99	0.69	0.85 (0.40–1.84)	0.49	0.69 (0.23–2.03)
LA	36.12	36.34	0.71	1.03 (0.88–1.20)	0.16	1.14 (0.95–1.37)
BMI	23.35	24.14	0.70	0.97 (0.82–1.14)	0.81	0.98 (0.80–1.19)
Male	50.00	7	0.96	1.03 (0.30–3.05)	0.79	0.81 (0.17–3.81)
Diabetes	12	0	0.39	0.04 (0.00–67.4)	0.98	0.00 (0.00–)
Hypertension	49	4	0.39	0.58 (0.17–1.99)	0.22	0.37 (0.07–1.84)
CHD	15	1	0.46	0.46 (0.06–3.61)	0.74	0.67 (0.06–7.39)
Smoke	29	4	1.00	1.00 (0.29–3.41)	0.36	2.32 (0.38–14.21)
CKD	14	1	0.51	0.52 (0.06–3.92)	0.89	1.21 (0.09–16.76)
LVEF	68.56	68.76	0.50	1.03 (0.94–1.14)	0.32	1.07 (0.94–1.21)

*AI, ablation index; Ata, atrial tachyarrhythmia; LA, left atrium; BMI, body mass index; CHD, coronary heart disease; CKD, chronic kidney disease; LVEF, left ventricular ejection fraction.*

### Ablation Time and Complications

The procedure was successfully completed in all 90 patients, the procedure duration (172.4 ± 41.1 min vs. 176.2 ± 36.2 min vs. 168.3 ± 46.7 min, *p* = 0.37) and fluoroscopy time (21.17 ± 5.59 min vs. 18.42 ± 5.18 min vs. 18.42 ± 5.62 min, *p* = 0.71) reached no significant difference, but the ablation time was significantly reduced in the low AI group (group C) compared with the other two groups (47.8 ± 14.6 min. vs. 47.0 ± 15.6 min vs. 36.6 ± 8.9 min, *P* < 0.001), and there was no statistical difference between groups A and B. Four patients had pericardial effusion, 3 in group A, 1 in group B, and 0 in group C (10% vs. 3.3% vs. 0%, *P* = 0.32) (group A + B vs. group C: 6.7% vs. 0%, *P* = 0.30), all pericardial effusions were treated with pericardiocentesis (aspiration of fluid volume: 100–200 ml) without surgery. There was no steam pop in all procedures. One arteriovenous fistula occurred in group A, the patient was asymptomatic and without hematoma at the puncture site. Other complications, such as death, thromboembolic events, major bleeding, PV stenosis, phrenic nerve palsy, or symptoms suggestive of esophageal injury were not observed during the study period.

## Discussion

**FIGURE 5:**
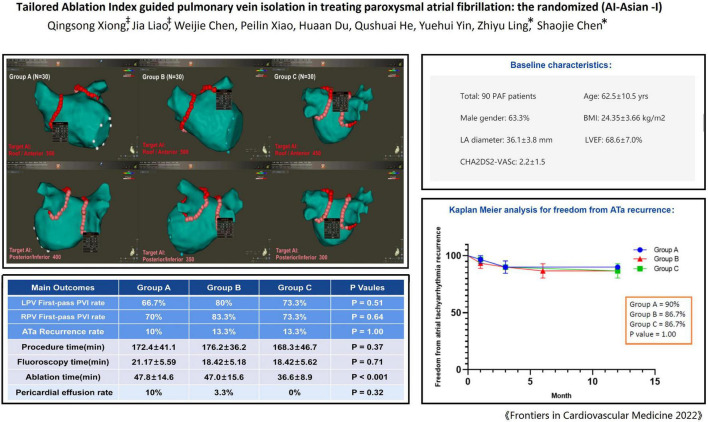
Graphic summary.

The main findings are summarized in Figure 5. In this randomized study, we compared different target AI guided PVI in treating paroxysmal AF. The overall rate of first-pass PVI was 73.3%, and the rate of first-pass PVI did not significantly differ between the three groups, also with similar gap rate during the procedural waiting time. Lower AI guided PVI (group C) was associated with significantly less ablation time than that in the other two groups, whereas higher AI guided PVI seemed to link a non-significant trend toward higher rate of pericardial effusion/tamponade, although the rate of overall complications was not different. One-year follow-up showed single-procedural similar freedom from ATa recurrence among the three groups. To the best of our knowledge, this is the first randomized study investigating different target ablation index to guide pulmonary vein isolation in Asian population.

Previous studies showed that AI guided AF ablation may improve the procedural efficacy (18); however, studies to define optimal AI value remains limited. According to the anatomy, the thickness of atrial tissue is about 2–4 mm. *In vitro* experiments showed that, every 100 increase of the AI caused lesion-depth increase by 1 mm. Thus theoretically, the transmural lesion can be achieved when the AI reaches 200–400 depending on the thickness of atrial wall. From the literature, the mostly adopted target AI was around 500–550/350–400, mainly from European-American population, and such target AI led to a first-pass rate of PVI around 70–90% (19–24).

Notably, the patients enrolled in our study appeared to have significantly lower BMI (24 kg/m^2^) and smaller LA (36 mm). Consistent with previous studies, the overall rate of first-pass PVI in our study was 73.3%, and interestingly the rate of first-pass PVI did not significantly differ between the three groups, indicating that relatively lower AI guided ablation might be effective to achieve procedural PVI among Asian population. Such assumption seemed to be further supported by the similar procedural gap rate (even during the waiting time) and the similar ATa recurrence rate during follow-up.

Indeed, the 70–80% first-pass PVI observed in our study was not high. A relatively lower power (35W) adopted in our study may play a role since it has been known that high-power ablation may form wider ablation lesion. Second, the target inter-lesion distance of 5 mm was employed in our study, as previous randomized study found that a relatively wider inter-lesion distance (5–6 mm) was associated with significantly lower rate of first-pass PVI as compared with that with closer inter-lesion distance (3–4 mm), when using conventional power ablation for PVI (25).

In general, effective lesion formation with short ablation time indicates an efficient procedure, or even may be safe procedure. Longer ablation time can be potentially associated with increased risk of procedural complication. In our study, four (4/90) patients had pericardial effusion after the AI guided PVI without evidence of notable steam-pop, all pericardial effusion occurred in higher AI groups (3 in group A, 1 in group B), and no pericardial effusion occurred in group C. More specifically, all pericardial effusions were detected after the PVI during routine echocardiography, and all these patients had additional ablation due to no first-pass isolation. For the four patients who had pericardial effusion: three were female, one male patient had known renal dysfunction, all four patients were in older age (mean 70 years old) with relatively low BMI (mean 24 kg/m^2^), procedural ACTs were between 300 and 350, and no audible steam-pop was noticed during the procedures. The four patients with pericardial effusion were treated with pericardiocentesis (aspiration of fluid volume: 100–200 ml), without needing surgery. These observations may indicate that, (1) pericardial effusion could still occur regardless of steam-pop; (2) repeated or additional ablation to achieve the target AI thereby to close the gap sites due to failure of first-pass isolation appears to be a risk factor of pericardial effusion; (3) the recommended target AI derived from the European-American population might not be necessarily suitable for Asian population, and should be performed with cautions, e.g., in small, older, female patients.

In our study, lower AI guided ablation (group C) was associated with significantly less ablation time. However, we did not count overall procedure and fluoroscopic time for every patient because some patients also had concomitant electrophysiological examination, and some patients had concomitant coronary angiography. Although this was a randomized study, as an initial validation study, the sample size in each group was rather small. We could only investigate the procedural efficacy of the different AI guided PVI, and the long-term durable PVI could not be assessed in the present study. The ATa recurrence was only assessed by Holter-ECG, without continuous heart rhythm monitoring, thus some episodes of ATa recurrences may not be diagnosed in all the three groups. Conventional power instead of high power was used, thus our result cannot be generalized to high power ablation strategy. Due to unavailability our patients could not receive esophageal temperature monitoring during ablation. As preventive strategy, the atrial esophageal anatomical relationship was carefully assessed by pre-procedural cardiac CT or MRI and integrated in the 3D mapping system, energy delivery at those potential adjacent sites were maximally avoided. In addition, all patients received 4 weeks preventive PPI therapy after the ablation, and no patients had symptoms or signs suggestive of esophageal injury during clinical follow-up.

## Conclusion

This randomized study shows that, a relatively lower target AI guided AF ablation may be similarly effective to achieve PVI with significantly reduced ablation time and obtain similar clinical outcome in treating paroxysmal atrial fibrillation in Asian population.

## Data Availability Statement

The original contributions presented in the study are included in the article/supplementary material, further inquiries can be directed to the corresponding author/s.

## Ethics Statement

The study was approved by Ethics Committee of Second Affiliated Hospital of Chongqing Medical University. The patients/participants provided their written informed consent to participate in this study.

## Author Contributions

ZL: study design. QX, JL, WC, PX, HD, QH, YY, and ZL: data collection. QX and JL: data analysis. QX, ZL, and SC: first draft. QX, JL, WC, PX, HD, QH, YY, ZL, and SC: review and approval. ZL and SC were co-mentors for the dissertation. SC dedicated intellectual contribution to the dissertation, interpreted the study results, and made critical revision of the manuscript. All authors contributed to the article and approved the submitted version.

## Conflict of Interest

SC has been invited as consultant to Biosense Webster, Boston Scientific, and declares no financial funding with regard to the content of the manuscript. The remaining authors declare that the research was conducted in the absence of any commercial or financial relationships that could be construed as a potential conflict of interest.

## Publisher’s Note

All claims expressed in this article are solely those of the authors and do not necessarily represent those of their affiliated organizations, or those of the publisher, the editors and the reviewers. Any product that may be evaluated in this article, or claim that may be made by its manufacturer, is not guaranteed or endorsed by the publisher.
